# Cost-effectiveness of acupuncture for irritable bowel syndrome: findings from an economic evaluation conducted alongside a pragmatic randomised controlled trial in primary care

**DOI:** 10.1186/1471-230X-12-149

**Published:** 2012-10-24

**Authors:** Eugena Stamuli, Karen Bloor, Hugh MacPherson, Helen Tilbrook, Tracy Stuardi, Sally Brabyn, David Torgerson

**Affiliations:** 1 Department of Health Sciences, University of York, York, YO10 5DD, UK

**Keywords:** Acupuncture, Irritable bowel syndrome, Economic evaluation, Health technology assessment, Quality of life

## Abstract

**Background:**

There is insufficient evidence to determine whether acupuncture is a cost-effective treatment for irritable bowel syndrome. The objective of this study is to assess the cost-effectiveness of acupuncture as an adjunct to usual care versus usual care alone for the treatment of Irritable Bowel Syndrome (IBS).

**Methods:**

Cost-utility analysis conducted alongside a pragmatic, multicentre, randomised controlled trial. 233 patients with irritable bowel syndrome were randomly allocated to either acupuncture plus usual care, or usual care alone. Cost-effectiveness outcomes are expressed in terms of incremental cost per quality adjusted life year (QALY) at one year after randomisation. Costs were estimated from the UK National Health Service perspective for a time horizon of one year. Cost-utility ratios were estimated based on complete case analysis for the base case analysis, where only patients with available EQ-5D and cost data were included. Sensitivity analyses comprised a multiple imputation approach for missing data and a subgroup analysis for the more severe cases of IBS.

**Results:**

The base case analysis showed acupuncture to be marginally more effective than usual care (gain of 0.0035 QALYs, 95% CI: -0.00395 to 0.0465) and more expensive (incremental cost of £218 per patient (95% CI: 55.87 to 492.87) resulting in an incremental cost-effectiveness ratio of approximately £62,500. Sensitivity analysis using multiple imputation for missing data resulted in acupuncture appearing less effective and more costly than usual care, so usual care is dominant. Subgroup analysis selecting the most severe cases of IBS (Symptom Severity Score of over 300) suggested that acupuncture may be a cost-effective treatment option for this group, with a cost-per-QALY of £6,500.

**Conclusions:**

Acupuncture as an adjunct to usual care is not a cost-effective option for the whole IBS population; however it may be cost-effective for those with more severe irritable bowel syndrome.

**Trial registration:**

Current Controlled Trials ISRCTN08827905

## Background

Population-based studies have demonstrated that the prevalence rates of irritable bowel syndrome range from 2.1% to 22% [1]. The main symptoms are abdominal pain or discomfort, altered form, frequency and passage of stools, and abdominal distension. IBS affects all age groups and it is believed that factors such as familial aggregation, early life events, diet and psychosocial conditions might drive the development of the disease [1].

The disease is not life-threatening, but leads to significant impairment of health related quality of life, which reflects physical role limitations as well as pain and a lower perception of general health [2]. As a consequence, patients with IBS are more likely than those without to have impaired daily routines, relationships, social lives and emotional status [3]. IBS causes high level of absenteeism and impairs workplace functioning; the magnitude of impairment is directly related to severity and frequency of bowel symptoms [4].

In the UK, patients with gastrointestinal symptoms are primarily seen by a GP. Of those diagnosed with IBS, approximately 20% are referred to a gastroenterologist or general physician and 9% to a surgeon [4]. Several studies have estimated the NHS costs associated with IBS [3,5]. They have concluded that the direct and indirect costs associated with IBS are substantial. The cost driver is usually higher hospital inpatient episodes experienced by patients with IBS. The loss due to impaired productivity in the UK has also been estimated to be significant [6] mainly due to missed days of work which have been estimated to be between 1.53 [3] and 1.7 days per month [7].

A Cochrane review supports the finding that conventional treatments for IBS are rarely effective in managing all of the symptoms associated with IBS [8]. Hence, there is a tendency of patients increasingly turning to complementary and alternative medicine [9], one of them being acupuncture. There is insufficient evidence to determine whether acupuncture is an effective treatment for IBS [10], and no evidence of cost-effectiveness from an NHS perspective. This paper reports the relative cost-effectiveness of acupuncture for IBS, assessed as part of a pragmatic randomized controlled trial undertaken in the UK [11,12].

## Methods

### Study design, setting, participants and interventions

Details and clinical results of this trial are reported elsewhere (clinical paper – *under submission*). Briefly, 233 participants were recruited from primary care in the English NHS into a pragmatic randomised controlled trial of acupuncture for IBS. 116 were randomised to receive a short course of traditional acupuncture plus usual GP care and 117 to receive usual GP care alone. The treatment course of the acupuncture arm included up to 10 sessions over a three month period.

The economic analysis assesses the relative cost-effectiveness of adding acupuncture sessions to usual care compared with usual care alone for the treatment of IBS. A cost-utility analysis approach was adopted, where utility data are based on the EQ-5D questionnaire collected at three-monthly intervals from baseline to 12 months. The UK NHS perspective was taken [13], where only costs directly linked to the NHS budget were included. The time frame of the analysis was one year; hence, no discounting was applied to costs or outcomes.

The outcomes of the analysis are presented as incremental costs per quality adjusted life year (QALY). Uncertainty around the estimates is presented as the probability that acupuncture added to usual care is cost-effective for a range of values that decision makers are willing to pay for gaining one additional QALY, illustrated using as cost-effectiveness acceptability curves (CEACs).

### Resource use data and outcome measures

NHS costs (GP and nurse visits, emergency and elective hospitalisations, outpatient attendances and contact with other NHS professionals) were included. Resource use data were collected from GP records. Data were collected for 15 months, which included three months before randomisation and the one year follow-up period of the trial. The prior period was used to assess the baseline consumption of health care resources. All contacts with health care professionals were recorded, not just those relating to IBS, but where IBS was documented in the patient notes this was recorded by the researcher. This allows the estimation of cost-effectiveness based on the disease specific resource consumption in addition to the overall consumption during the trial period. In addition, data were collected on the total number of acupuncture sessions attended by each patient allocated to the intervention arm.

For the outcome data, the EQ-5D instrument [14] was used to measure and value patients’ health states. This was collected at baseline (at the point of enrolment of the participants in the trial) and at three, six, nine and 12 months post-randomisation. Patients completed the EQ-5D instrument via a postal questionnaire. EQ-5D captures health on five three-level domains: no problems, moderate problems or severe problems in mobility, self care, usual activities, pain-discomfort, and anxiety-depression, resulting in 243 possible health states.

EQ-5D scores were converted to utility scores by using the social tariff based on preferences of the UK general population [15]. To calculate QALYs, the utility scores at each of the five time points were plotted and the area under the curve was estimated. This approach takes into account both the utility score at each time point and the duration of time between them, assuming that utility changes are linear from one time point to the next.

### Unit costs

Unit costs were identified for four types of activities: those provided in primary care (GP and practice nurse), hospital based activity (outpatient clinics, emergency and elective admissions), other NHS professionals (hospital or community based) and acupuncture sessions. Information on unit costs was extracted from three sources: Unit costs of health and social care [16], NHS reference costs [17] and NHS choices website [18]. Unit costs (at 2010 prices) are illustrated in Table 1.

**Table 1:** Unit costs of health care services

**Item [source]**	**Unit**	**Cost**	**Notes**
**GP and practice nurse**			
GP [16]	Per surgery consultation lasting between 11.7 minutes and 17.2 minutes	£44.5	Page 167
			Average of £36 (short visit) and £53 (long visit)
Nurse (GP practice) [16]	Per consultation	£12	Page 164
**Hospital based activity (outpatient clinics, emergency and elective admissions)**			
Outpatient activity [17]	Unit of activity	£98	Outpatient Attendances Data
Elective admissions [17]	Unit of activity	£1612	Elective inpatient HRG
			The unit cost was derived as a weighted average of all the activities that had duration of 1 day (similar to the duration of elective admissions for IBS dataset)
Emergency admissions (for duration of stay 1 nights, 2 days) [17]	Event	£134	Accident and Emergency Services: leading to Admitted**.** The unit cost was derived as a weighted average of all the activities.
Emergency admissions (for duration of stay 0 nights, 1 day) [17]	Event	£103	Accident and Emergency Services: not Leading to Admitted. The unit cost was derived as a weighted average of all the activities.
**Other NHS professionals (hospital or community based)**			
Physiotherapist Occupational therapist [16]	Clinic visit	£17	Page 151
Chiropodist/Podiatrist [16]	Clinic visit	£11	Page 154
Counsellor [16]	Surgery consultation	£71	Page 78
Community psychiatric nurse (CPN) [16]	One hour of face-to-face contact	£56	Page 160
Psychologist/Psychiatrist/ Psychotherapist [16]	One hour of client contact	£81	Page 155. Assume psychiatrist and psychotherapist as well
Mental health [16]	Per hour of face-to-face contact	£70	Page 184. Assume involvement of community mental health team
Radiology/X-ray [17]	Unit of activity	£27	Outpatient Attendances Data
			Diagnostic imaging
Ophthalmology/Eye care/Retinopathy [17]	Unit of activity	£80	Outpatient Attendances Data
Gynaecology/Colposcopy [17]	Unit of activity	£112	Outpatient Attendances Data
Family planning [17]	Unit of activity	£59	Outpatient Attendances Data for
			Sexual and Reproductive Health Clinic (previously referred to as Family Planning Clinic)
Midwife [17]	Antenatal visit	£46	Community Midwifery Services: Visits
	Postnatal visit	£58	
Urology [17]	Unit of activity	£99	Outpatient Attendances Data
Medical Gastroenterology Sigmoidoscopy/Colonoscopy [17]	Unit of activity	£123	Outpatient Attendances Data
Neurology [17]	Unit of activity	£166	Outpatient Attendances Data
**Acupuncture sessions**			
Initial session [18]	Session	£47.5	Initial sessions usually cost between £35 and £60, and further sessions between £25 and £50.
Further session [18]	Session	£37.5	Unit costs calculated as average of two values

### Analysis

EQ-5D scores and resource use data were missing for some patients. The base case analysis was conducted as a “complete case analysis” where only patients with available utility scores at all points and available costs were included.

An additional analysis was conducted by including all the patients that returned EQ-5D questionnaires for at least one time-point and had at least partial information on resource use. Missing EQ-5D scores were imputed based on a group of variables (age, treatment allocation and the utility scores of the patients at any time-point). The cost data for the patients who had partial information (e.g. they had left the GP surgery), were inflated based on the time for which the information was available. This was done in a linear fashion, assuming the cumulative costs would increase proportionally for the rest of the trial duration. For each of the scenarios, two analyses were conducted; first by including all costs irrespective of their nature, and second by including only the IBS-related costs.

The differences in cost data are presented unadjusted and adjusted for baseline costs; the latter being either total or only IBS-related costs, depending on the nature of the analysis. QALY data were adjusted for baseline EQ-5D scores as recommended by Manca et al [19].

For the cost-effectiveness analysis, the mean differences in costs and effects were estimated by using seemingly unrelated regressions and the 95% confidence intervals around those were estimated using bias corrected and accelerated (BCA) bootstrap methods. Analyses were conducted using STATA Version 10.1.

A pre-specified subgroup analysis was conducted based on baseline Symptom Severity Scores (SSS) [20]. Patients were grouped as mild (SSS = 75–175), moderate (SSS = 175-300) and severe (SSS =300+). Subgroup analysis was conducted using only complete cases and only for the total health care resource usage (not on the IBS-only costs).

## Results

### Missing data

Data were completely missing for 6 patients randomized in the acupuncture and 20 in the usual care arm. Of those, GP records could not be traced for 3 patients as they had left the surgery and no further information was available for them. 9 patients withdrew from the trial; hence their GP records were not accessed. The records of 14 patients were not available from the GP registry.

There was partial information on resource use for three patients (two in acupuncture and one in the usual care arm) as they left the GP surgery at some point after the trial initiation. This was at 8 and 11 months after the randomization date for two patients allocated in the acupuncture and one month after in the usual care arm.

There were missing utility scores for both arms of the trial. The number of missing utility scores, due to either missing questionnaires or missing domains in the EQ-5D questionnaire, was higher for the usual care arm for all the time-points. For the acupuncture, the range of missing utility scores was 2.6% at baseline to 18.1% at month nine. For the usual care arm, the rates of missing utility scores ranged from 2.6% at baseline to 30.8% at month six.

### Resource use

Table 2 presents levels of resource use based on the available trial data. During the trial, participants in the acupuncture arm had a higher mean number of GP visits (6.7 vs. 5.9) and nurse visits (2.5 vs. 2) compared with usual care. Usual care patients had a higher number of emergency admissions but a lower number of outpatient clinic contacts compared with the acupuncture arm.

**Table 2:** Resource use

	**Acupuncture (n = 110)**	**Usual care (n = 97)**	**Mean difference (95%CI)**
	**mean (SD)**	**mean (SD)**	
**GP visits**			
Baseline visits	1.67 (2.46)	1.65 (1.87)	0.02 (−0.58 to 0.63)
IBS related visits at baseline	0.22 (0.53)	0.36 (1.00)	−0.14 (−0.36 to 0.07)
Visits during the trial	6.69 (8.69)	5.89 (5.74)	0.81 (−1.24 to 2.85)
IBS related visits during the trial	0.87 (1.83)	0.84 (1.62)	0.04 (−0.44 to 0.51)
**Practice nurse**			
Baseline visits	0.42 (0.99)	0.46 (0.74)	−0.05 (−0.29 to 0.20)
IBS related visits at baseline	0.05 (0.39)	0.02 (0.14)	0.03 ( −0.06 to 0.11)
Visits during the trial	2.48 (5.21)	1.99 (2.93)	0.49 (−0.69 to 1.67)
IBS related visits during the trial	0.12 (0.63)	0.05 (0.27)	0.07 (−0.07 to 0.20)
**Elective hospitalisations**			
Baseline	0.01 (0.10)	0.00 (0.00)	0.01 (−0.01 to 0.03)
IBS related at baseline	0.00 (0.00)	0.00 (0.00)	NA
During the trial	0.05 (0.21)	0.05 (0.22)	0.01 (−0.07 to 0.05)
IBS related during the trial	0.01 (0.10)	0.00 (0.00)	0.01 (−0.01 to 0.028)
**Emergency hospitalisations**			
Baseline	0.04 (0.19)	0.09 (0.29)	−0.06 (−0.12 to 0.01)
IBS related at baseline	0.01 (0.10)	0.00 (0.00)	0.01 (−0.01 to 0.03)
During the trial	0.25 (0.72)	0.32 (0.80)	−0.07 (−0.28 to 0.13)
IBS related during the trial	0.03 (0.16)	0.00 (0.00)	0.03 (−0.01 to 0.06)
**Outpatient clinics**			
Baseline	0.40 (1.00)	0.29 (0.66)	0.11 (−0.12 to 0.35)
IBS related at baseline	0.09 (0.35)	0.04 (0.20)	0.05 (−0.03 to 0.13)
During the trial	1.49 (2.30)	1.45 (2.45)	0.04 (−0.62 to 0.69)
IBS related during the trial	0.31 (0.98)	0.24 (0.83)	0.08 (−0.18 to 0.32)
**Other NHS contacts**			
Baseline	0.07 (0.58)	0.06 (0.37)	0.01 (−0.12 to 0.13)
IBS related at baseline	0 (0)	0.01 (0.09)	−0.01 ( −0.03 to 0.01)
During the trial	0.35 (1.10)	0.48 (2.13)	−0.13 (−0.58 to 0.31)
IBS related during the trial	0.02 (0.18)	0 (0)	0.02 (−0.02 to 0.05)
**Acupuncture sessions**	9.18 (2.18)	NA	NA

### Costs

A summary of costs based on the available trial data is presented in Table 3.

**Table 3:** Mean costs based on available data

**Type of costs**	**Acupuncture (n = 110)**	**Usual care (n = 97)**	Mean difference (95% CI)^*^
	**Mean(SD) (£ sterling)**	**Mean(SD) (£ sterling)**	
Total baseline costs (for 3 months prior to trial)	144 (264)	123 (128)	21 (−38 to 79)
Total IBS baseline costs (for 3 months prior to trial)	20( 49)	21 (54)	−1 (−15 to 13)
Health care costs during trial (excluding acupuncture)	600 (725)	574 (728)	26 (−174 to 225)
IBS-related costs during trial (excluding acupuncture)	91 (245)	61 (123)	30 (−24 to 84)
Acupuncture costs during trial	339 (98)	0 (0)	339 (321 to 356)
Total health care costs during trial (including acupuncture)	940 (740)	574 (728)	366 (164 to 567)
Total IBS-related costs during trial (including acupuncture)	431 (269)	61(122)	370 (311 to 429)

* Difference is not adjusted for baseline costs.

Acupuncture patients had higher baseline costs compared to the usual care patients (£144 vs. £123) as well as during the trial (£600 vs. £574). The latter does not include the cost of acupuncture sessions. IBS related costs during the trial were also higher for the acupuncture patients (£91 vs. £61), again without including the cost of acupuncture sessions. Total costs during the trial (acupuncture sessions plus health care costs) were higher for the acupuncture group compared with usual care patients (£940 vs. £574), a difference of £366.

### Health states

Mean utility scores by time point are presented in Table 4. The baseline scores were lower for the acupuncture arm. At month 3, utility scores were higher for the usual care patients compared to those receiving acupuncture (0.78 vs. 0.74, difference adjusted for baseline scores of 0.0351) while at months 6 and 9 acupuncture was marginally better or no different from the usual care arm. At month 12, utility scores for the acupuncture patients were marginally worse than those for the usual care group. None of the differences reached conventional levels of statistical significance.

**Table 4:** Summary of utility scores based on EQ-5D at each time point (available cases)

**Variable**	**Acupuncture**		**Usual care**		Adjusted mean difference (95% CI)^*^
**Utility**	**N**	**Mean (SD)**	**N**	**Mean (SD)**	
Baseline	113	0.7121 (0.220)	114	0.7335 (0.1972)	−0.0214(−0.0759 to 0.0332)
Month 3	106	0.7405 (0.219)	82	0.7810 (0.1614)	−0.0351(−0.0833 to 0.0132)
Month 6	99	0.7528 (0.246)	81	0.7430 (0.2070)	0.0125(−0.0442 to 0.0692)
Month 9	95	0.7389 (0.229)	88	0.7379 (0.2234)	0.0008(−0.0546 to 0.0562)
Month 12	99	0.7342 (0.257)	90	0.7439 (0.2024)	−0.0128(−0.0682 to 0.0425)

* The difference at 3, 6, 9, 12 moths is adjusted for baseline EQ-5D scores.

### Cost-effectiveness analysis

#### Base case analysis

The complete case analysis, based on all costs (IBS and non IBS related) demonstrated that acupuncture results in a QALY gain at 12 months of 0.0035 (BCA 95% CI: -0.0395 to 0.0465) for unadjusted and 0.0033 (BCA 95% CI: -0.0398 to 0.0462) adjusted for baseline costs (see Table 5). Acupuncture is more expensive than usual care resulting in incremental costs of £218.50 per patient (BCA 95% CI: 55.87 to 492.87) for unadjusted and £230.78 (BCA 95% CI:-34.52 to 496.08) for adjusted results. This leads to an incremental cost-effectiveness ratio of £62,429 (£69,933) per QALY gained based on unadjusted (adjusted) estimates.

**Table 5:** Full group and subgroup analyses (based on the complete case approach)

	**Acupuncture Mean (SD) (n = 77)**	**Usual care Mean (SD) (n = 53)**	**Difference unadjusted (BCA 95% CI)**	Difference adjusted^*^(BCA 95% CI)
	**Costs**			
Total costs at baseline (full group)	113 (256)	126 (138)		
Mild	72 (146)	169 (138)		
Moderate	134 (329)	119 (126)		
Severe	95 (122)	130 (170)		
Total costs during trial (full group)	869 (680)	650 (833)	**218.50 (−55.17 to 492.18)**	**230.78 (−34.52 to 496.08)**
Mild	900 (936)	404 (264)	***496.46 (−244.24 to 1237.17***	***590.57 (−51.58 to 1232.72)***
Moderate	876 (722)	698 (1014)	***178.61 (−251.54 to 608.77)***	***164.59 (−229.41 to 558.60)***
Severe	774 (403)	576 (355)	***197.68 (−29.68 to 425.03)***	***231.87 (−11.68 to 475.42)***
	**Outcomes**			
QALYs (full group)	0.7534 (0.1846)	0.7365 (0.1870)	**0.0035 (−0.0389 to 0.0458)#**	**0.0033 ( −0.0398 to 0.0462)**
Mild	0.7650 (0.2431)	0.8356 (0.1034)	***−0.0406 (−0.2038 to 0.1227)***	***−0.0399 (−0.2053 to 0.1256)***
Moderate	0.7822 (0.1720)	0.7752 (0.1541)	***−0.0103 (−0.0586 to 0.0380)***	***−0.0107 (−0.0596 to 0.0382)***
Severe	0.7049 (0.1885)	0.6683 (0.2363)	***0.0310 (−0.0576 to 0.1197)***	***0.0309 (−0.0588 to 0.1206)***

Note: baseline SSS missing for 1 Acupuncture patient and 3 Usual care patients.

#The difference in QALYs is always adjusted for baseline EQ-5D scores.

*The difference in costs is adjusted for baseline costs.

**Figure 1:**
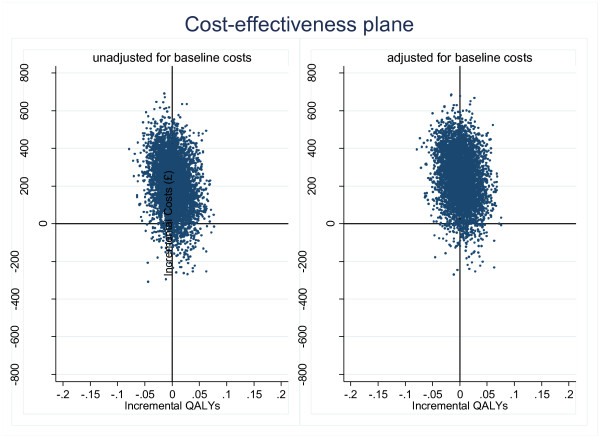
Scatter plot of differences in costs and QALYs (complete case analysis- all costs included).

**Figure 2:**
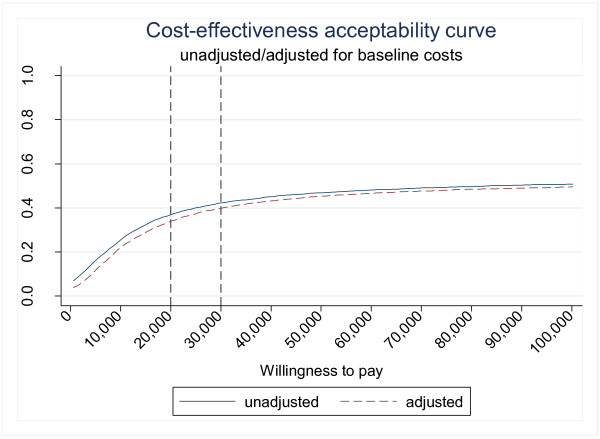
Cost-effectiveness acceptability curve (complete case analysis- all costs included).

Figure 1 shows the incremental costs and QALYs. Figure 2 presents the probability that acupuncture is cost-effective for a range of maximum values that decision makers may be willing to pay for an additional QALY gained. At a value of £30,000 per QALY gained, often stated to be the borderline for the NHS [13], acupuncture has a 40% probability of being cost effective. The analysis based on IBS-related costs only led to similar conclusions i.e. acupuncture is associated with higher costs and marginally better outcomes. The ICER in this case is around £137,000 per QALY for both adjusted and unadjusted results.

#### Multiple imputation analysis

The analysis based on the multiple imputation of missing outcome and cost data demonstrated that acupuncture is less effective and more costly (including all IBS and non-IBS related costs). Acupuncture costs £365.75 (BCA 95% CI: 362.91 to 368.60) for unadjusted for baseline values costs and £341.52 (BCA 95% CI: 339.00 to 344.03) for the adjusted costs, more than usual care. The difference in outcomes (acupuncture – usual care) is −0.0064 (BCA 95% CI: -0.0069 to −0.0059), leading to usual care being a dominant option. The same conclusion is reached when the analysis is based on only IBS-related costs.

#### Subgroup analysis

The subgroup analysis demonstrated that usual care is dominant for mild and moderate cases of IBS patients, as acupuncture appears more costly and less effective than usual care. For severe cases of IBS (with an SSS symptom score of over 300), acupuncture appears more effective, although also more costly. The QALY gain is approximately 0.031 (−0.0588 to 0.1206) and incremental costs are between £198 and £232 (−34.52 to 496.08) (unadjusted and adjusted results). These lead to an incremental cost-effectiveness ratio of £6,377 (£7,504) based on unadjusted (adjusted) results. The probability that acupuncture is cost-effective is slightly over 60% for willingness-to-pay values of £20,000 to £30,000 per QALY gained. This reflects the uncertainty around this result.

## Discussion

### Principal findings

Our base case analysis demonstrated that acupuncture is more costly and marginally more beneficial than the usual care alone. Incremental cost effectiveness ratios were within a range of £60,000 to £70,000 per QALY.

Sensitivity analysis resulted in usual care being dominant for mild and moderate cases, but a subgroup analysis suggested acupuncture could be a cost-effective option for the patients with more severe IBS, as defined by the IBS SSS score.

### Strengths and limitations

This study is the first economic evaluation of acupuncture for IBS patients based on a randomized controlled trial. The trial was pragmatic and the results have strong external validity and applicability to everyday clinical practice in the UK setting.

Missing data limits slightly the validity of the results. However, the analysis was based on two different scenarios of handling missing data to test the robustness of the conclusions. The sensitivity analysis did reverse the direction of difference in outcomes, making the case that acupuncture is not a cost-effective option to be adopted in everyday practice even stronger.

Another potential limitation of the study arises from the exclusion of medication costs in the economic analysis. As data on health resources consumption was extracted from the GP records, tracking down the medication usage was problematic. However, previous research has shown that the cost drivers are usually high inpatient episodes and consultations with the GP [3,5]; hence, the exclusion of medication costs is very unlikely to have changed the conclusions of this study.

Subgroup analysis, suggesting the possibility of acupuncture being cost-effective in more severe IBS patients, is informative but preliminary. The uncertainty around this result reflects the low sample size. There is a 60 per cent probability that treatment of this subgroup would meet NICE thresholds for cost-effectiveness.

### Implications for clinical practice

The lack of notable benefit in terms of health utility from the treatment drives the main conclusions of this analysis. This trial does not support the use of acupuncture for IBS patients as an appropriate use of NHS resources. It is possible that acupuncture may be a cost-effective treatment option for patients with severe IBS, a group that is difficult to treat, with poor quality of life [21,22].

### Recommendations for future research

The subgroup analysis suggested that acupuncture may be cost-effective for patients with severe IBS. This finding can assist in hypothesis building for further examination and analysis. For example, a trial which is powered to detect differences in outcomes for this subgroup of patients might be pertinent.

In addition, an economic model to extrapolate long term costs and effects would also be helpful in more clearly demonstrating the cost effectiveness or otherwise of acupuncture for this group of severe IBS patients, since the utilisation of health care resources over the long term could then be incorporated directly into the economic modelling approach and accounted for in the economic analysis.

## Conclusions

Our principal finding is that acupuncture as an adjunct to usual care is not a cost-effective option for the whole IBS population. Preliminary analysis suggests that it it may be a cost-effective option for those with more severe irritable bowel syndrome.

### Ethics approval

We received ethics approval from the York NHS Research Ethics Committee (08/H1311/66) in 2008.

## Competing interests

HM has a part-time clinical practice that includes acupuncture.

## Authors’ contributions

ES was the trial health economist who conducted the cost effectiveness analyses. KB provided advice on all aspects of the economic analysis and its interpretation. HM conceived the study and was the lead applicant and principal investigator for the trial. HT was the Trial Co-ordinator. TS and SB contributed to the trial management. DT provided support and advice in his role as Director of the York Trials Unit. All authors have had full access to all of the data (including statistical reports and tables) in the study and can take responsibility for the integrity of the data and the accuracy of the data analysis, and have read and approved the final manuscript.

## Funding body

This article presents independent research commissioned by the UK National Institute for Health Research (NIHR)’s Research for Patient Benefit programme (PB-PG-0407-13241). The views expressed in this publication are those of the authors and not necessarily those of the NHS, the NIHR or the UK Department of Health.

## Sponsor

The University of York, in its role as sponsor for the study, had no input into study design; in the collection, analysis, and interpretation of data; in the writing of the report; and in the decision to submit the article for publication.

## Pre-publication history

The pre-publication history for this paper can be accessed here:

http://www.biomedcentral.com/1471-230X/12/149/prepub
